# Poster Session II - A259 INVESTIGATING THE ROLE OF MICROBIAL PROTEIN METABOLISM ACROSS DEFINED MICROBIOTA MOUSE MODELS AND INFLAMMATORY CONDITIONS

**DOI:** 10.1093/jcag/gwaf042.259

**Published:** 2026-02-13

**Authors:** K Beaudoin, B Barbosa da, Luz Confetti, P Muppidi, L Rondeau, L Li, A Caminero

**Affiliations:** McMaster University, Hamilton, ON, Canada; McMaster University, Hamilton, ON, Canada; McMaster University, Hamilton, ON, Canada; McMaster University, Hamilton, ON, Canada; University of Alberta, Edmonton, AB, Canada; McMaster University, Hamilton, ON, Canada

## Abstract

**Background:**

Inflammatory bowel disease (IBD) involves dysregulated inflammation shaped by environmental factors, including diet and the gut microbiota. IBD prevalence is rising in Western countries with diets high in animal protein, fat, and sugar. Diet-microbiota interactions are thought to play a significant role in intestinal inflammation, and microbial composition and function are known to be altered in IBD. Our lab recently showed that high protein diets (HPD) worsen colitis by activating the mechanistic target of rapamycin (mTOR) and suppressing autophagy. Branched-chain amino acids (BCAAs) and arginine, abundant in HPD, are potent mTOR activators. Therefore, we hypothesize that microbial protein metabolism is altered after inflammation and produces bioactive compounds that enhance mTOR activation.

**Aims:**

Determine how gut microbiota and intestinal inflammation influence dietary protein metabolism and mTOR activation.

**Methods:**

Specific-pathogen-free (SPF), altered Schaedler flora (ASF; stable murine microbiota of 8 bacterial strains), and germ-free (GF) C57BL/6 mice received a control diet (CD;14% protein; n = 5/group) or HPD (40%; n = 6/group) for two weeks to study the importance of the microbiota on protein metabolism and mTOR activation. To study the role of inflammation on microbial protein metabolism, colitis was induced in SPF C57BL/6 mice with 3% dextran sulfate sodium (DSS) for 5 days, followed by 2 days of water. mTOR activation was assessed by p-S6 expression, a downstream target of mTOR, using immunohistochemistry in colon and small intestine tissue. Fecal samples were analyzed using LC-MS metabolomics to quantify total amino acids, BCAAs, and related metabolites.

**Results:**

Gut microbiota composition strongly shaped protein metabolism. On the CD, GF, ASF, and SPF mice had distinct metabolite profiles; SPF mice had higher amino acid-derived metabolites (spermine, spermidine, para-cresol). After DSS, SPF mice had increased BCAAs (valine, isoleucine) and arginine versus baseline, and higher putrescine, spermine, spermidine, and cadaverine. GF mice displayed greater colonic p-S6 expression than ASF and SPF mice on CD. Under HPD conditions, GF mice showed increased small-intestinal mTOR activation relative to ASF and SPF mice. Inflammation increased colonic mTOR activation.

**Conclusions:**

The gut microbiota regulates dietary protein metabolism and modulates mTOR activity, as microbiota-defined differences influence inflammatory signalling. Intestinal inflammation elevates colonic amino acids with mTOR-activating potential. Future work will extend HPD-focused metabolomics in mouse models and human IBD cohorts.

**Funding Agencies:**

CCC, CIHR

